# Supplementary material for: Effects of Primary Aldosteronism and Different Therapeutic Modalities on Glucose Metabolism
Source: J Clin Med. 2019 Dec 12;8(12):2194. doi: 10.3390/jcm8122194 (PMC6947343; doi:10.3390/jcm8122194)
Supplement: Supplementary file 1 [file jcm-08-02194-s001.pdf]

Effects of Primary Aldosteronism and Different Therapeutic Modalities on Glucose Metabolism

Mi Kyung Kwak [1], Jee Yang Lee [2], Beom-Jun Kim [2], Seung Hun Lee [2,*] and Jung-Min Koh[2,*]

[1] Division of Endocrinology and Metabolism, Hallym University Dongtan Sacred Heart Hospital, Dongtan, 7, Keunjaebong-gil, Hwaseong, Gyeonggi-do, 18450, Republic of Korea; creamtea38@gmail.com (M.K.K.)

[2] Division of Endocrinology and Metabolism, Asan Medical Center, University of Ulsan College of Medicine, 88, Olympic-ro 43-gil, Songpa-gu, Seoul 05505, Korea; asanmedicalamc@gmail.com (J.Y.L.); umkbj0825@amc.seoul.kr (B-J.K.); hun0108@amc.seoul.kr (S.H.L.); jmkoh@amc.seoul.kr (J-M.K.)

* Correspondence: hun0108@amc.seoul.kr; Tel.: +82-2-3010-5666 (S.H.L.); jmkoh@amc.seoul.kr; Tel.: +82-2-3010-3247 (J-M.K.)

The Supplemental data includes Tables S1-S6 and Figure S1.

Table S1. Glucose status in patients with PA without SH and matched controls.

	Matched control (N = 609)	PA without SH (N = 245)	*p*
Pre-diabetes, N (%)	**173 (24.7%)**	**90 (36.7%)**	**0.002**
DM, N (%)	**90 (12.9%)**	**46 (18.8%)**	**0.024**
Hyperglycemia, N (%)	**263 (37.6%)**	**136 (55.5%)**	**<0.001**

Up to three controls were enrolled per participant with PA. They were individually matched for sex, age (±1 year), and BMI (±0.5 kg/m^2). The hyperglycemia was defined as DM or pre-diabetes.
Significant results (p<0.05) are indicated in bold.
BMI, body mass index; DM, diabetes mellitus; PA, primary aldosteronism; SH, subclinical hypercortisolism.

Table S2. Glucose status in drug-naïve patients with PA and matched controls.

	Matched control (N = 225)	PA (N = 81)	*p*	Matched control (N = 205)	PA without SH (N = 73)	*p*
FPG (mg/dL)	103.5 ± 25.5	102.2 ± 14.9	0.672	103.2 ± 25.8	101.3 ± 14.0	0.560
Insulin (pmol/L)	**10.2 ± 5.7**	**7.3 ± 4.3**	**<0.001**	**10.3 ± 5.8**	**7.3 ± 4.4**	**<0.001**
HOMA-β	**108.1 ± 63.9**	**74.9 ± 52.8**	**<0.001**	**109.6 ± 65.5**	**76.1 ± 54.3**	**<0.001**
HOMA-IR	**2.6 ± 1.6**	**1.9 ± 1.2**	**<0.001**	**2.6 ± 1.7**	**1.9 ± 1.3**	**<0.001**

Up to three controls were enrolled per participant with PA. They were individually matched for sex, age (±1 year), and BMI (±0.5 kg/m^2).
Significant results (p<0.05) are indicated in bold. Data are expressed as the mean ± standard deviation.
FPG, fasting plasma glucose; HOMA-β, homeostasis model assessment–β-cell function index; HOMA-IR, homeostasis model assessment–insulin resistance index; PA, primary aldosteronism; SH, subclinical hypercortisolism

Table S3. The association of PRA, PAC, ARR, or PAC after SIT with HOMA-IR and HOMA-β in drug-naïve patients with PA

	HOMA-β (N = 81)				HOMA-IR (N =81)			
Variable	β*	SE	Beta†	*p*	β	SE	Beta	*p*
PRA (ng/mL/h)								
Model 1	0.533	7.280	0.008	0.942	0.195	0.167	0.130	0.248
Model 2	−1.506	7.076	−0.023	0.832	0.120	0.154	0.080	0.438
Model 3	−1.668	7.190	−0.026	0.817	0.120	0.157	0.080	0.447
PAC (ng/dL)								
Model 1	−25.980	13.486	−0.212	0.058	**−0.760**	**0.309**	**−0.267**	**0.016**
Model 2	−23.041	12.885	−0188	0.078	**−0.666**	**0.278**	**−0.234**	**0.019**
Model 3	−22.718	13.360	−0185	0.093	**−0.672**	**0.288**	**−0.236**	**0.023**
ARR, (ng/dL)/(ng/mL/h)								
Model 1	−5.344	6.005	−0.100	0.376	**−0.280**	**0.136**	**−0.225**	**0.044**
Model 2	−3.547	5.840	−0.066	0.545	−0.215	0.126	−0.172	0.092
Model 3	−3.207	5.939	−0.060	0.591	−0.211	0.128	−0.169	0.104
PAC after SIT (ng/dL)								
Model 1	**−37.039**	**15.318**	**−0.281**	**0.018**	−0.436	0.350	−0.149	0.217
Model 2	**−47.038**	**14.584**	**−0.357**	**0.002**	−0.631	0.333	−0.217	0.063
Model 3	**−49.367**	**15.181**	**−0.375**	**0.002**	−0.643	0.349	−0.221	0.070

*Non-standardized coefficient. †Standardized coefficient.
The Enter method was applied to this model.
Significant results ($P<0.05$) are indicated in bold. PRA, PAC, ARR, and PAC after SIT were log-transformed because of their skewed distributions.
Model 1: unadjusted model; Model 2: adjusted for sex, age, and BMI; Model 3: adjusted for current smoking status, alcohol intake (≥3 units/day), and regular outdoor exercise (≥30 min/day), in addition to the variables included in Model 2.
ARR, PAC/PRA ratio; BMI, body mass index; HOMA-β, homeostasis model assessment–β-cell function index; HOMA-IR, homeostasis model assessment–insulin resistance index; PA, primary aldosteronism; PAC, plasma aldosterone concentration; PRA, plasma renin activity; SIT, saline infusion test.

Table S4. Baseline characteristics of patients with PA, classified according to therapy.

	ADX (N = 178)	MRA (N = 108)	*p*
Men, N (%)	84 (47.2%)	61 (56.5%)	0.161
Age (years), mean ± SD	52.4 ± 10.5	54.9 ± 11.5	0.063
BMI (kg/m^2), mean ± SD	25.5 ± 3.4	25.7 ± 4.1	0.680
Current smoker, N (%)	29 (16.3%)	23 (21.3%)	0.365
Alcohol intake ≥3 U/day, N (%)	26 (14.6%)	23 (21.3%)	0.196
Regular exercise ≥30 min/day, N (%)	**23 (12.9%)**	**28 (25.9%)**	**0.009**
SBP (mmHg), mean ± SD	140.3 ± 18.2	139.3 ± 19.0	0.653
DBP (mmHg), mean ± SD	86.9 ± 12.2	85.1 ± 11.8	0.221
Hypertension, N (%)	178 (100.0%)	108 (100.0%)	>0.999
K^+ (mEq/L), mean ± SD	**3.6 ± 0.6**	**4.3 ± 2.3**	**0.003**
Creatinine (mg/dL), mean ± SD	0.8 ± 0.2	0.8 ± 0.2	0.767
eGFR (mL/min), mean ± SD	97.1 ± 30.6	97.4 ± 29.6	0.944
PRA (ng/mL/h) , mean ± SD	0.4 ± 1.0	0.9 ± 3.3	0.096
PAC (ng/dL), mean ± SD	**38.1 ± 25.4**	**25.6 ± 12.8**	**<0.001**
ARR, (ng/dL)/(ng/mL/h), mean ± SD	**200.6 ± 200.2**	**112.3 ± 198.5**	**<0.001**
PAC after SIT (ng/dL) , mean ± SD	**25.4 ± 18.4**	**11.4 ± 5.2**	**<0.001**

Significant results (p<0.05) are indicated in bold. ADX, adrenalectomy; ARR, PAC/PRA ratio; BMI, body mass index; BP, blood pressure; DBP, diastolic blood pressure; GFR, glomerular filtration rate; MRA, mineralocorticoid receptor antagonist; PA, primary aldosteronism; PAC, plasma aldosterone concentration; PRA, plasma renin activity; SBP, systolic blood pressure; SIT, saline infusion test.

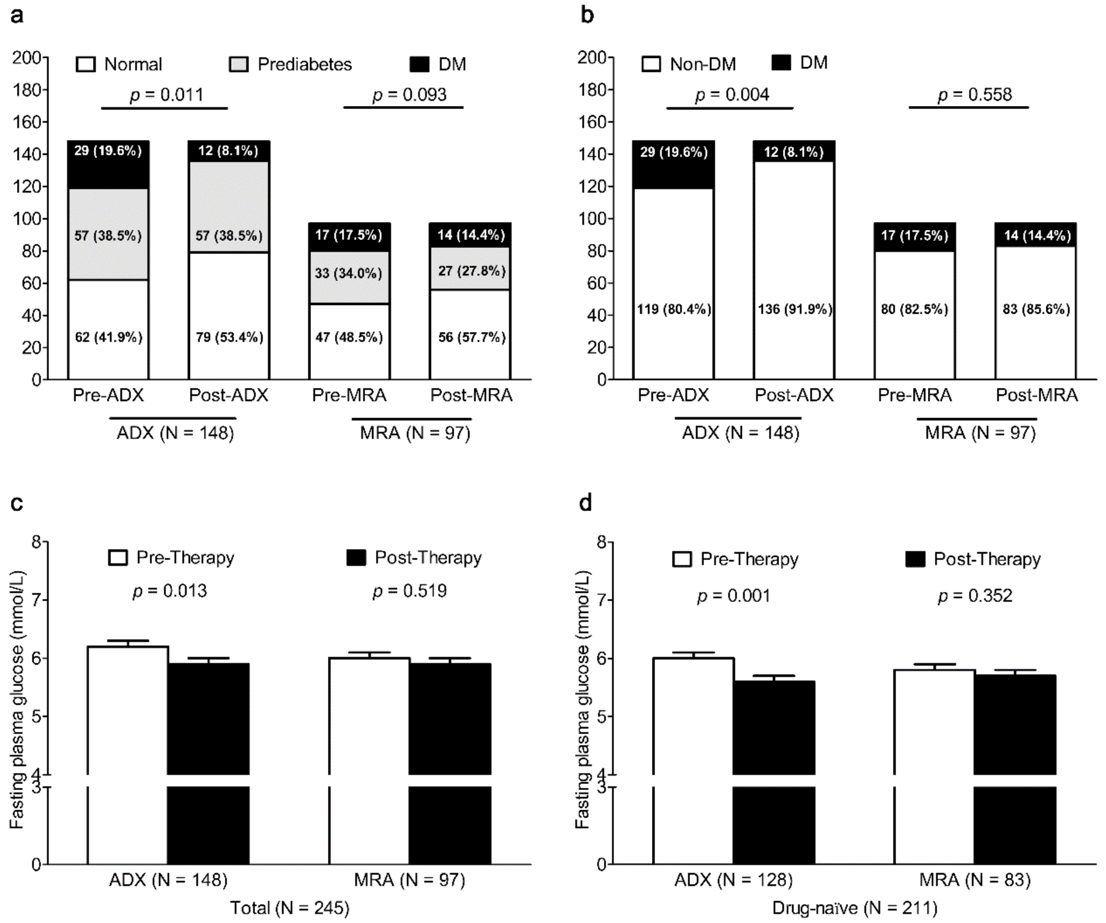

Figure S1. Change in glucose status and FPG in PA patients without SH after ADX or MRA therapy. Change in glucose status (normal, prediabetes, and DM in Figure S1a; non-DM and DM in Figure S1b) in PA patients without SH after ADX or MRA therapy. Change in FPG in PA patients without SH (Figure S1c) and that in anti-diabetic therapy naïve PA patients without SH (Figure S1d) after ADX or MRA therapy. ADX, adrenalectomy; DM, diabetes mellitus; FPG, fasting plasma glucose; MRA, mineralocorticoid receptor antagonist; SH, subclinical hypercortisolism

Table S5. Likelihood of improvement in glucose status following ADX vs. MRA therapy in PA patients with similar ranges of plasma aldosterone concentration, after an intravenous saline infusion test

	OR (95% CI)	*p*
Model 1		
MRA therapy	Ref.	
ADX	**2.11 (1.17–3.82)**	**0.013**
Model 2		
MRA therapy	Ref.	
ADX	**2.09 (1.14–3.82)**	**0.017**
Model 3		
MRA therapy	Ref.	
ADX	**2.22 (1.20–4.13)**	**0.012**
Model 5		
MRA therapy	Ref.	
ADX	**2.23 (1.16–4.27)**	**0.016**

Significant results ($p<0.05$) are indicated in bold.
Model 1: unadjusted model; Model 2; adjusted for sex, age, and BMI; Model 3: adjusted for current smoking status, alcohol intake (≥3 units/day), and regular outdoor exercise (≥30 min/day), in addition to the variables included in Model 2; Model 5: adjusted for plasma aldosterone concentration after intravenous saline infusion test, in addition to the variables included in Model 3.
Improvement in glucose status was defined as a change in glucose status from diabetes mellitus to pre-diabetes or normal glucose tolerance, or from pre-diabetes to normal glucose tolerance, or by the reduction of anti-diabetic medication.
ADX; adrenalectomy, BMI; body mass index; MRA, mineralocorticoid receptor antagonist

Table S6. Likelihood of improvement in glucose status following ADX in unilateral PA patients vs. MRA therapy in bilateral PA patients

	OR (95% CI)	*p*
Model 1		
MRA therapy	Ref.	
ADX	**1.81 (1.03–3.18)**	**0.038**
Model 2		
MRA therapy	Ref.	
ADX	**1.81 (1.02–3.21)**	**0.043**
Model 3		
MRA therapy	Ref.	
ADX	**1.90 (1.05–3.42)**	**0.033**
Model 5		
MRA therapy	Ref.	
ADX	**1.99 (1.01–4.06)**	**0.049**

Significant results ($p<0.05$) are indicated in bold.
Unilateral PA patients: 148 patients with unilateral APA and 20 patients with UAH
Bilateral PA patients: 66 patients with BAH and 10 patients with bilateral APA
Model 1: unadjusted model; Model 2; adjusted for sex, age, and BMI; Model 3: adjusted for current smoking status, alcohol intake (≥3 units/day), and regular outdoor exercise (≥30 min/day), in addition to the variables included in Model 2; Model 5: adjusted for plasma aldosterone concentration after intravenous saline infusion test, in addition to the variables included in Model 3.
Improvement in glucose status was defined as a change in glucose status from diabetes mellitus to pre-diabetes or normal glucose tolerance, or from pre-diabetes to normal glucose tolerance, or by the reduction of anti-diabetic medication.
ADX; adrenalectomy, APA, aldosterone-producing adenoma; BAH, bilateral adrenal hyperplasia; BMI; body mass index; MRA, mineralocorticoid receptor antagonist; UAH, unilateral adrenal hyperplasia
